# Bulk-Processed Plasmonic Plastic Nanocomposite Materials for Optical Hydrogen Detection

**DOI:** 10.1021/acs.accounts.3c00182

**Published:** 2023-06-23

**Authors:** Iwan Darmadi, Ida Östergren, Sarah Lerch, Anja Lund, Kasper Moth-Poulsen, Christian Müller, Christoph Langhammer

**Affiliations:** † Department of Physics, Chalmers University of Technology, 412 96 Göteborg, Sweden; ‡ Department of Chemistry and Chemical Engineering, Chalmers University of Technology, 412 96 Göteborg, Sweden; § Institute of Materials Science of Barcelona, ICMAB-CSIC, Bellaterra, 08193 Barcelona, Spain; ∥ Catalan Institution for Research and Advanced Studies ICREA, Pg. Lluís Companys 23, 08010 Barcelona, Spain; ⊥ Department of Chemical Engineering, Universitat Politècnica de Catalunya, EEBE, Eduard Maristany 10−14, 08019 Barcelona, Spain

## Abstract

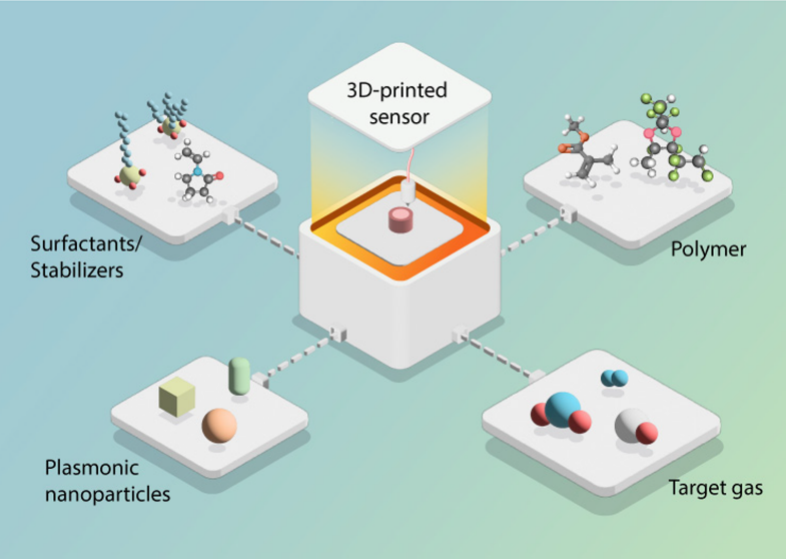

Sensors are ubiquitous, and their importance is only going to increase across many areas of modern technology. In this respect, hydrogen gas (H_2_) sensors are no exception since they allow mitigation of the inherent safety risks associated with mixtures of H_2_ and air. The deployment of H_2_ technologies is rapidly accelerating in emerging energy, transport, and green steel-making sectors, where not only safety but also process monitoring sensors are in high demand. To meet this demand, cost-effective and scalable routes for mass production of sensing materials are required. Here, the state-of-the-art often resorts to processes derived from the microelectronics industry where surface-based micro- and nanofabrication are the methods of choice and where (H_2_) sensor manufacturing is no exception.

In this Account, we discuss how our recent efforts to develop sensors based on plasmonic plastics may complement the current state-of-the-art. We explore a new H_2_ sensor paradigm, established through a series of recent publications, that combines (i) the plasmonic optical H_2_ detection principle and (ii) bulk-processed nanocomposite materials. In particular, plasmonic plastic nanocomposite sensing materials are described that comprise plasmonic H_2_-sensitive colloidally synthesized nanoparticles dispersed in a polymer matrix and enable the additive manufacturing of H_2_ sensors in a cost-effective and scalable way. We first discuss the concept of plasmonic plastic nanocomposite materials for the additive manufacturing of an active plasmonic sensing material on the basis of the three key components that require individual and concerted optimization: (i) the plasmonic sensing metal nanoparticles, (ii) the surfactant/stabilizer molecules on the nanoparticle surface from colloidal synthesis, and (iii) the polymer matrix. We then introduce the working principle of plasmonic H_2_ detection, which relies on the selective absorption of H species into hydride-forming metal nanoparticles that, in turn, induces distinct changes in their optical plasmonic signature in proportion to the H_2_ concentration in the local atmosphere. Subsequently, we assess the roles of the key components of a plasmonic plastic for H_2_ sensing, where we have established that (i) alloying Pd with Au and Cu eliminates hysteresis and introduces intrinsic deactivation resistance at ambient conditions, (ii) surfactant/stabilizer molecules can significantly accelerate and decelerate H_2_ sorption and thus sensor response, and (iii) polymer coatings accelerate sensor response, reduce the limit of detection (LoD), and enable molecular filtering for sensor operation in chemically challenging environments. Based on these insights, we discuss the rational development and detailed characterization of bulk-processed plasmonic plastics based on glassy and fluorinated matrix polymers and on tailored flow-chemistry-based synthesis of Pd and PdAu alloy colloidal nanoparticles with optimized stabilizer molecules. In their champion implementation, they enable highly stable H_2_ sensors with response times in the 2 s range and an LoD of few 10 ppm of H_2_. To put plasmonic plastics in a wider perspective, we also report their implementation using different polymer matrix materials that can be used for 3D printing and (an)isotropic Au nanoparticles that enable the manufacturing of macroscopic plasmonic objects with, if required, dichroic optical properties and in amounts that can be readily upscaled. We advertise that melt processing of plasmonic plastic nanocomposites is a viable route toward the realization of plasmonic objects and sensors, produced by scalable colloidal synthesis and additive manufacturing techniques.

## Key References

- Nugroho, F. A. A.; Darmadi, I.; Cusinato, L.; Susarrey-Arce, A.; Schreuders, H.; Bannenberg, L. J.; da Silva Fanta, A. B.; Kadkhodazadeh, S.; Wagner, J. B.; Antosiewicz, T. J.; Hellman, A.; Zhdanov, V. P.; Dam, B.; Langhammer, C. Metal–Polymer Hybrid Nanomaterials for Plasmonic Ultrafast Hydrogen Detection. *Nat. Mater.***2019**, *18* (5), 489–495.^1^ This study of polymer-coated PdAu alloy nanoparticle arrays showed that polymers accelerate and enhance plasmonic H_2_ sensor response, lower the limit of detection, prevent sensor deactivation by selective molecular filtering, and enable sub-1 s response at 1 mbar H_2_.
- Pekkari, A.; Say, Z.; Susarrey-Arce, A.; Langhammer, C.; Härelind, H.; Sebastian, V.; Moth-Poulsen, K. Continuous Microfluidic Synthesis of Pd Nanocubes and PdPt Core–Shell Nanoparticles and Their Catalysis of NO_2_ Reduction. *ACS Appl. Mater.Interfaces***2019**, *11* (39), 36196–36204.^2^ This work presents a continuous flow synthesis route for the synthesis of uniformly sized Pd nanocubes and PdPt core–shell nanoparticles in a single-phase microfluidic reactor, which enables rapid formation of shaped nanoparticles with a reaction time of 3 min.
- Darmadi, I.; Stolaś, A.; Östergren, I.; Berke, B.; Nugroho, F. A. A.; Minelli, M.; Lerch, S.; Tanyeli, I.; Lund, A.; Andersson, O.; Zhdanov, V. P.; Liebi, M.; Moth-Poulsen, K.; Müller, C.; Langhammer, C. Bulk-Processed Pd Nanocube–Poly(methyl methacrylate) Nanocomposites as Plasmonic Plastics for Hydrogen Sensing. *ACS Appl. Nano Mater.***2020**, *3* (8), 8438–8445.^3^ This study introduces bulk-processed and 3D-printed plasmonic plastic H_2_ sensors comprised of hydrogen-sensitive plasmonic Pd nanocubes mixed with a poly(methyl methacrylate) matrix that enable H_2_ detection in CO containing synthetic air and retain full functionality after 50 weeks.
- Östergren, I.; Pourrahimi, A. M.; Darmadi, I.; da Silva, R.; Stolaś, A.; Lerch, S.; Berke, B.; Guizar-Sicairos, M.; Liebi, M.; Foli, G.; Palermo, V.; Minelli, M.; Moth-Poulsen, K.; Langhammer, C.; Müller, C. Highly Permeable Fluorinated Polymer Nanocomposites for Plasmonic Hydrogen Sensing. *ACS Appl. Mater.Interfaces***2021**, *13* (18), 21724–21732.^4^ This work demonstrates the use of amorphous Teflon AF, compounded with colloidal Pd nanoparticles prepared by highly scalable continuous flow synthesis, as matrix for bulk-processed plasmonic H_2_ sensors no longer limited by H_2_ diffusion through the matrix.

## 1 Introduction

Nanoplasmonic systems exploiting localized surface plasmon resonance (LSPR) have to the largest extent been developed based on nanofabricated or self-assembled arrangements of metal nanoparticles and nanostructures on surfaces. Over the years, such systems have proven an ideal platform for the realization of a wide range of technologies, including plasmonic sensors,^5^ photocatalysts,^6^ photovoltaic devices,^7^ plasmonic lasers,^8^ and optical metamaterials.^9^ In particular, sensors, in their most prominent application area of the life sciences, have been implemented in a plethora of designs geared toward the detection of biological analytes with point-of-care diagnostics, affordable test kits, and single-molecule detection being some core concepts that have driven this development.^10−13^ Similarly, plasmonic sensor platforms in a multitude of surface-based designs have been developed for measuring contaminations in water and food,^14^ as well as for detecting gaseous species,^15^ where the detection of H_2_ is the most widely explored and developed application^1,15−18^ and where other gaseous species, such as NO_2_,^19−23^ CO,^22^ and CO_2_,^24^ have been successfully targeted as well. For an overview of existing H_2_ sensor technologies and their corresponding strengths and weaknesses, we refer to our recent review of this topic.^17^

Over the years, additive manufacturing has emerged as a new paradigm for the creation of functional 3D objects.^25^ It delivers improved performance, access to complex geometries, and simplified fabrication by building 3D objects layer by layer. From a materials perspective, additive manufacturing most commonly uses neat materials, such as metals or polymers. However, recently, more complex systems have come into focus due to their potential to enable new applications. Here, so-called nanocomposites comprised of nanoparticles dispersed in a host matrix are particularly attractive since the material properties can be engineered at the nanoscale.^26^

Projecting the prospects of additive manufacturing onto nanoplasmonics reveals highly interesting opportunities in terms of scalable plasmonic material synthesis and processing routes for cost-effective device integration that have the potential to induce a paradigm shift in the field, as predicted by Haring et al.^27^ Simultaneously, 3D printing had been identified as a new concept for the manufacturing of sensors in general.^28^ Inspired by these developments, we have explored the potential of replacing surface-based plasmonic nanoparticle arrangements used widely for sensor applications with bulk-processed “plasmonic plastics”. This new class of nanocomposites is comprised of colloidal metal nanoparticles with a sensor function dispersed in a polymer matrix that facilitates the additive manufacturing of plasmonic (sensor) devices and simultaneously functions as a molecular filter, which improves sensor selectivity and enables operation in chemically challenging environments.

In this Account, which is based on a series of papers that we have published during the last 4 years,^1−4,29−32^ we will discuss our key discoveries, as well as the rational design and optimization of plasmonic plastic nanocomposites and their constituents, i.e., colloidal plasmonic nanoparticles and the polymer matrix, with respect to bulk processing, optical/plasmonic properties, and application in the area of optical H_2_ sensing. In developing this story, we focus on materials design based on a fundamental understanding of the limiting factors for the targeted application. Furthermore, we demonstrate how synthesis and processing of plasmonic plastic nanocomposites can be scaled to the kilogram range using flow synthesis of colloidal nanoparticles in combination with large-scale polymer compounding infrastructure.

## 2 Conceptual Approach to Plasmonic Plastics for Gas Sensing

**Figure 1:**
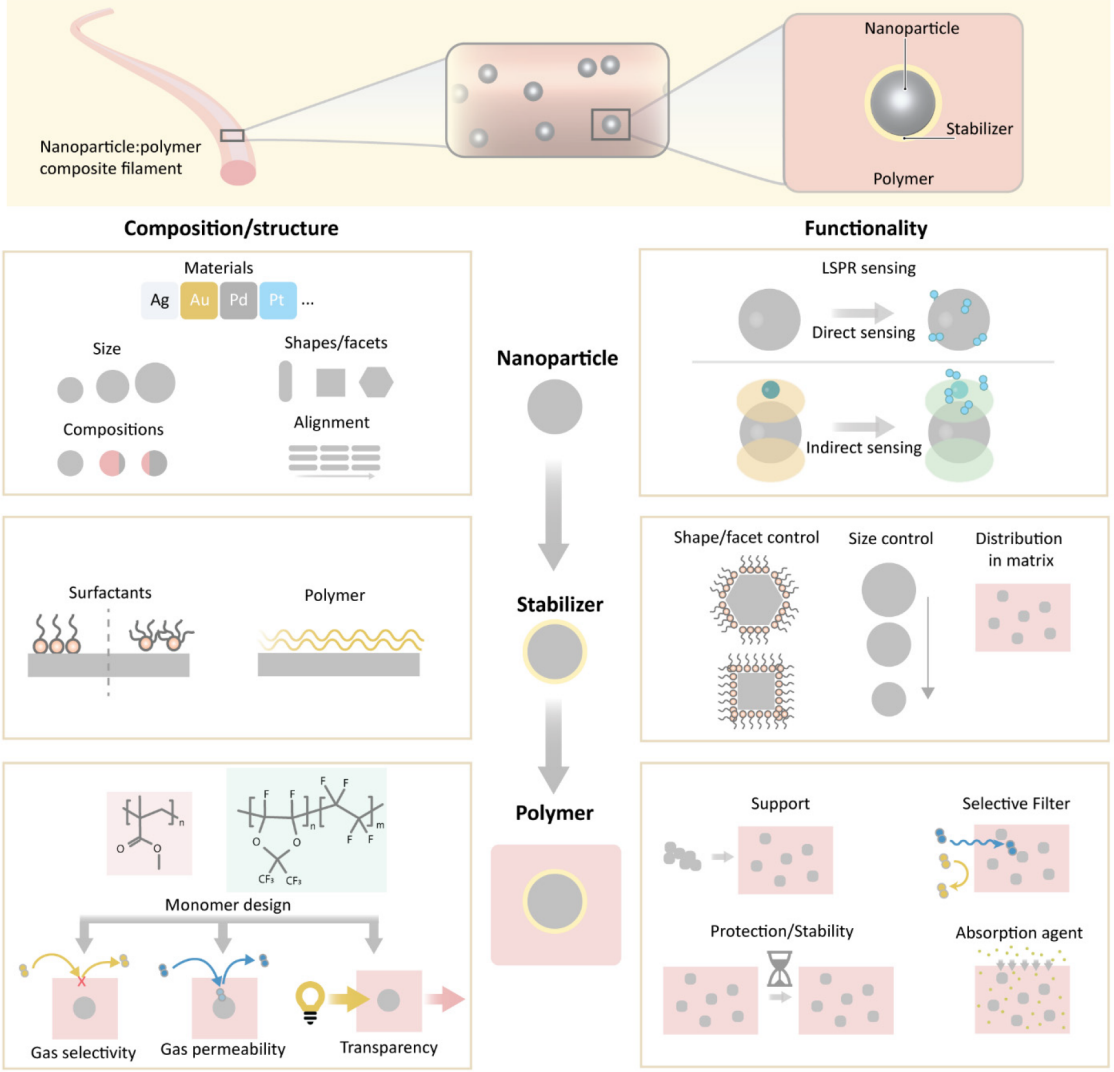
Schematic depiction of the “plasmonic plastic” nanocomposite material concept. Plasmonic plastics comprise three key components: (i) plasmonic metal nanoparticles, (ii) surfactant/stabilizer molecules on the nanoparticle surface, and (iii) polymer matrix, which can be tailored regarding composition/structure and functionality.

The concept of plasmonic plastic nanocomposite materials for additive manufacturing of the active material in gas sensors in general and in H_2_ sensors in particular exploits a material system with three key components that have to be understood and optimized individually and in concert from a composition/structure and functionality perspective: (i) the plasmonic metal nanoparticles, (ii) the surfactant molecules present on nanoparticle surfaces, and (iii) the polymer matrix (Figure 1).

Different metals can be chosen to prepare nanoparticles for different sensing functionalities. To guide this selection, we can distinguish between direct and indirect plasmonic sensing schemes.^33−35^ In direct sensing, the nanoparticles constitute both the chemically active sensing material and the plasmonically active signal transducer. Well-established examples are H_2_-absorbing Pd^34^ or (surface) oxidizing Al^36^ and Cu^37^ nanoparticles. In indirect sensing, these two functions are separated in space and executed by two different materials where one of them is a (inert and usually Au or Ag) plasmonic nanoparticle probe and the second one is a closely adjacent sensing material that undergoes chemical change.^19,38−40^ Depending on the targeted sensing function and environment, nanoparticle size and shape can be rationally selected to steer both the spectral position of the plasmonic resonance^41^ and the abundance of specific surface sites that control the surface chemistry.^42^ Furthermore, their composition can be manipulated at the atomic level by forming nanoalloys with the purpose of engineering both optical^43−45^ and chemical^29,46,47^ properties. A final aspect is the alignment of anisotropic nanoparticles, such as nanorods, since this will significantly affect the optical properties of a composite material due to the existence of different plasmonic modes along the short and long rod axes, respectively.^48^

The second key component is the surfactant/stabilizer molecules on the colloidal nanoparticle surface that are necessary to prevent aggregation in solution (Figure 1) and promote shape-specific particle growth.^49^ The noble metal nanoparticles of interest here are usually decorated with either cationic molecules based on quaternary ammonium salts, e.g., CTAB (cetyltrimethylammonium bromide), CTAC (cetyltrimethylammonium chloride), and TOAB (tetraoctylammonium bromide), or polymers such as poly(vinylpyrrolidone) (PVP). At the same time, the presence of such ligands significantly alters the surface chemical properties of metal nanoparticles^50^ and their hydrogen sorption properties.^30^ In addition, the selection of surfactant/stabilizer along with the used solvent may influence the nanoparticle distribution in the polymer host.

The third key component is the polymer matrix (Figure 1). The choice of the polymer repeat unit and molecular weight, the processing parameters, and the resulting nano- and microstructure determine the dispersion of nanoparticles, as well as the optical and gas transport properties. In the case of semicrystalline polymers, the spherulite size impacts optical transparency (clarity and haze) in the spectral range of interest, while crystallinity and crystal size influence the gas transport. Amorphous polymers, in contrast, readily feature a high degree of optical transparency (provided the polymer is fully dense) and are void of crystallites that can impede gas transport, which means that they are preferable for optical sensor applications. Gas diffusivity and selectivity of amorphous and glassy polymers that we have selected, such as poly(methyl methacrylate) (PMMA) and a Teflon AF-type fluoropolymer, are determined by the fractional free volume (FFV) and the solubility of the gas. The gas diffusivity, *D*, of a glassy polymer scales with the FFV according to *D* ∝ *e*^–*B*/FFV^, where *B* is a constant.^51^ Since Teflon AF tends to pack poorly in the glassy state,^52,53^ it features a significantly larger *D* = 2.3 × 10^–5^ cm^2^ s^–1^ for H_2_ at 30 °C than PMMA with *D* = 6.6 × 10^–7^ cm^2^ s^–1^.^4^ In comparison, semicrystalline polymers feature a much lower H_2_ diffusivity of, e.g., *D* = 5.9 × 10^–8^ cm^2^ s^–1^ in the case of poly(vinylidene fluoride) (PVDF). Importantly, the polymer matrix may be tailored to function as a molecular filter. In the case of glassy polymers, the selectivity between two gases depends on the product between the relative solubility and the diffusivity, with the latter scaling with the relative molecular volume of the two gases,^54^ meaning that a smaller analyte, such as H_2_, will more rapidly enter a polymer matrix compared with, e.g., CO.

## 3 Plasmonic Hydrogen Sensing

**Figure 2:**
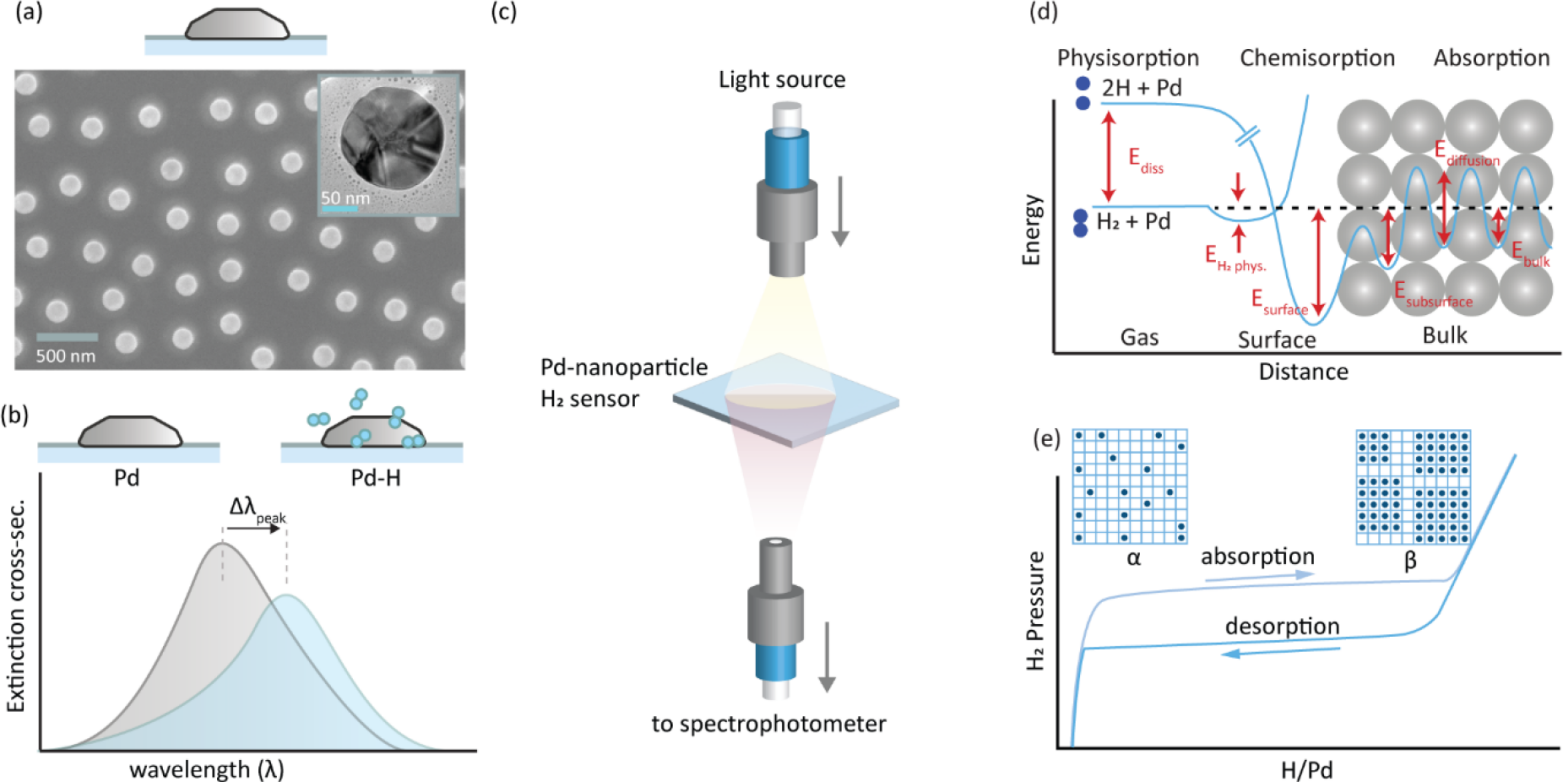
Nanoplasmonic H_2_ sensing based on Pd nanoparticles. (a) SEM image of a quasi-random Pd nanoparticle array nanofabricated onto a surface. The inset depicts a bright-field transmission electron microscopy (TEM) image of a single Pd nanoparticle that reveals a polycrystalline morphology obtained after thermal annealing at 500 °C. (b) Schematic of the plasmonic “peak” in the extinction spectrum of such a Pd nanoparticle array and how hydrogen sorption spectrally shifts the peak and its maximum, Δλ_peak_. (c) Schematic of an optical extinction measurement through a plasmonic sensor sample. (d) Schematic energy landscape of H_2_ dissociation on and H absorption into Pd. (e) Schematic of a pressure composition isotherm for H_2_ sorption in Pd that reveals the α-phase at low H_2_ pressures, the α- and β-coexistence region, the β-phase region at high H_2_ pressures, and the hysteresis between hydride formation and decomposition.

The first paper on plasmonic hydrogen sensing^55^ described nanofabricated quasi-random arrays of Pd nanoparticles in the 100 nm size range on a surface (Figure 2a). Distinct spectral shifts of the LSPR peak, Δλ_peak_, occurred upon absorption of hydrogen (Figure 2b). This enabled the measurement of optical pressure composition isotherms (Figure 2c). Later studies^33,34,47^ and first-principles calculations^56,57^ revealed direct proportionality between the H concentration in Pd and Δλ_peak_.

Mechanistically, the sensing process exploits the efficient hydrogen absorption into Pd at ambient conditions (Figure 2d). First, H_2_ molecules dissociate without activation barrier to occupy a chemisorbed state. Subsequently, the H species diffuse into interstitial subsurface positions crossing an activation barrier that defines the rate-limiting step, from which they diffuse interstitially into the lattice. For the reverse process, associative desorption of H_2_ is rate limiting. At low H_2_ (partial) pressure, *p*, H forms a solid solution (α-phase) with the Pd host and  according to Sieverts’ law. Upon increasing *p*, due to electronic and local strain field interactions between dissolved H species, the hydride (β-phase) nucleates and the system undergoes a first-order phase transformation that is characterized by a distinct plateau in the pressure–composition isotherm (Figure 2e). When the process is reversed and *p* is decreased, the hydride decomposes at a lower critical pressure. This thermodynamic hysteresis is the consequence of a lattice strain-induced energy barrier that has its origin in the volume expansion of the Pd host to accommodate H species.^58,59^ As discussed below, this hysteresis is problematic for sensor applications, and therefore, materials-engineering-based solutions have been developed.^17^ Pure metals other than Pd, such as Mg^60^ and Y,^61^ have also been used to control the optical properties of a nanoparticle-based plasmonic system by means of H_2_, and Hf-^62^ and Ta-based^63^ thin film systems have been developed for optical H_2_ sensing.

## 4 Understanding the Key Components of Plasmonic Plastics by 2D Model Systems

**Figure 3:**
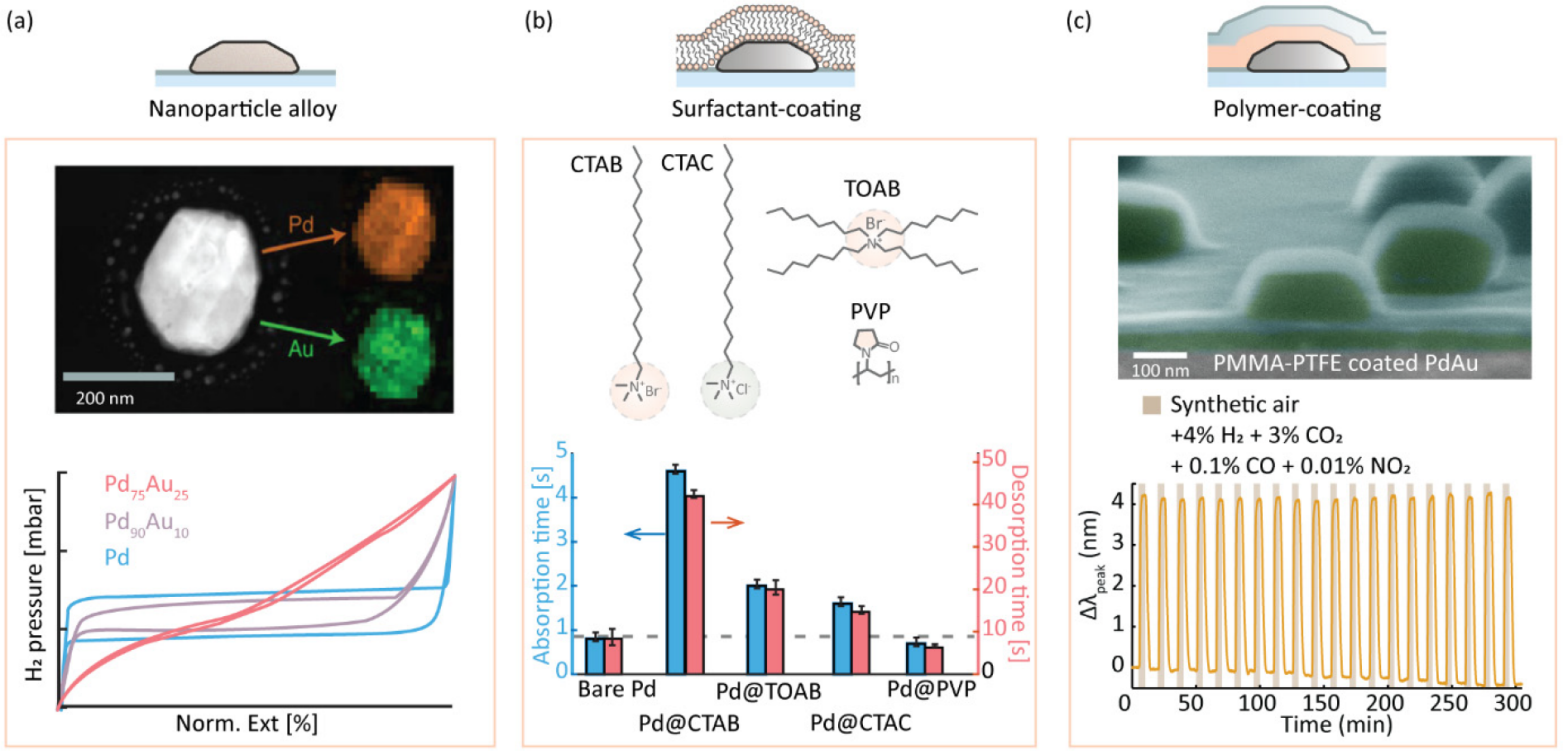
Two-dimensional model system investigation of the three key components of a plasmonic plastic. (a) Alloying Pd with noble metals eliminates hysteresis and enables linear response, as well as intrinsic deactivation resistance toward CO. Adapted from ref (65). Copyright 2015 American Chemical Society. (b) Cationic surfactant molecules CTAB, TOAB, and CTAC decelerate hydrogen sorption, whereas the polymer PVP accelerates it. Adapted from ref (30). Copyright 2020 American Chemical Society. (c) Thin film polymer (multi)layer coatings improve plasmonic hydrogen sensor performance by (i) lowering the LoD, (ii) accelerating the response, and (iii) enabling molecular filtering. Adapted with permission from ref (1). Copyright 2019 Nature Publishing Group.

To develop the foundation for rational plasmonic plastic nanocomposite design for H_2_ sensing, we separately investigated the role of the three key components based on 2D nanofabricated model systems on a surface (Figure 3). Starting with the nanoparticles, the intrinsic hysteresis of the Pd–H system can be eliminated by alloying with Au, Ag, or Cu (Figure 3a).^64^ Following this line, we have pioneered the nanofabrication and use of Pd–noble metal alloys for plasmonic hydrogen sensing applications^65−67^ and optimized their sensing metrics in synthetic air with trace amounts of CO, NO_2_, CH_4_, and CO_2_.^1,29,31,47,68^ We have found that the Pd_70_Au_30_ alloy system constitutes the best compromise between optical contrast and hysteresis-free and linear response and that the addition of Cu to the PdAu alloy generates an intrinsic deactivation resistance toward CO, with Pd_65_Au_25_Cu_10_ being the champion system. We also note that other alloy systems, such as PdCo,^69^ ZrY,^70^ and TaPd,^71^ show great promise for optical hydrogen sensing.

**Figure 4:**
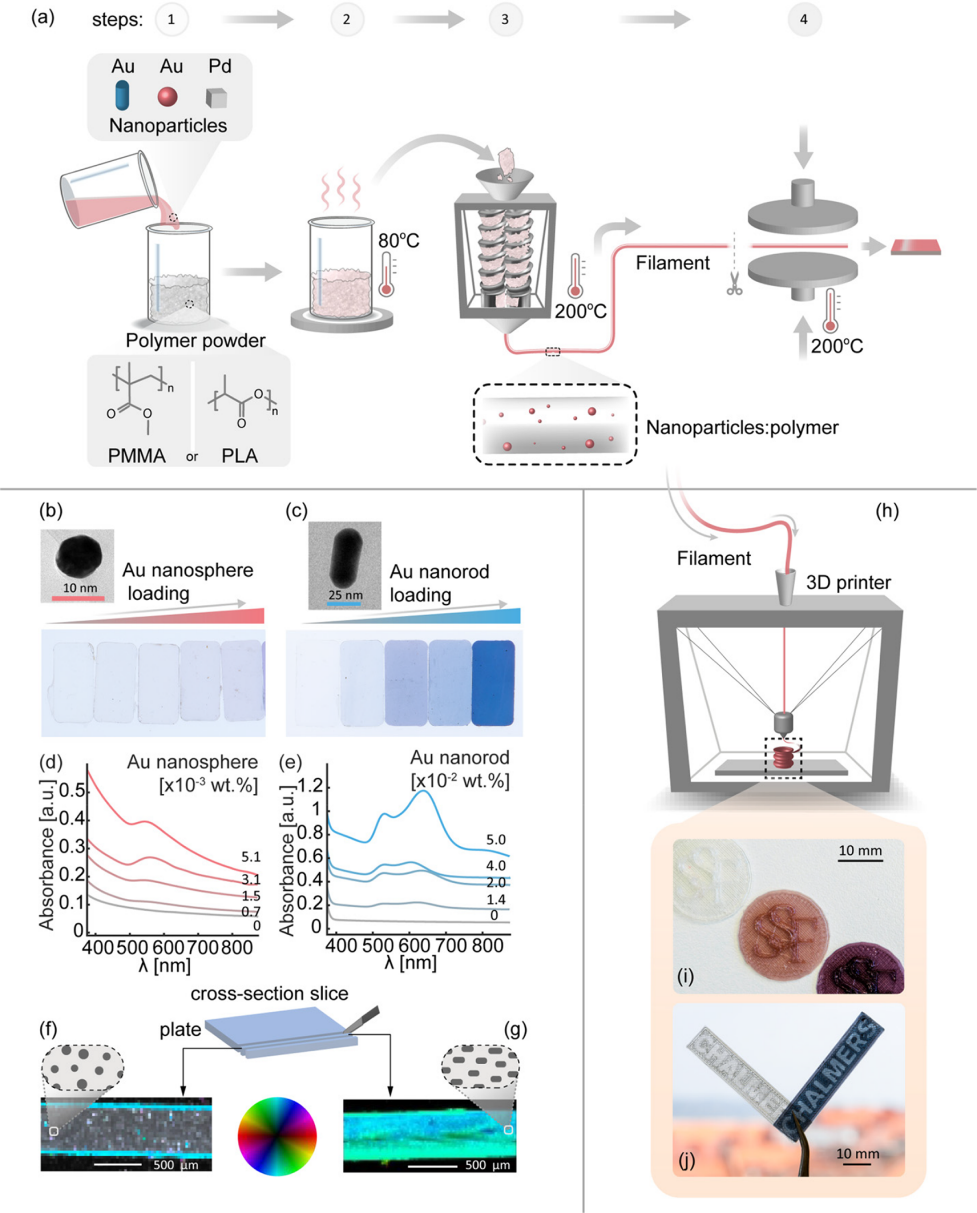
Au–nanosphere:PLA and Au–nanorod:PMMA plasmonic plastic nanocomposites. (a) Schematic of the processing sequence. (1) Mixing colloidal nanoparticle dispersion with polymer powder. (2) Drying. (3) Filament extrusion. (4) Melt pressing or 3D printing. Photographs of 500 μm thick (b) Au–nanosphere:PLA and (c) Au–nanorod:PMMA melt-pressed plates with increasing particle loading and 1 cm × 2 cm dimensions. Insets show TEM images of an Au nanosphere in the PLA and an Au nanorod in the PMMA matrix taken after the entire processing sequence. UV–vis spectra of the (d) Au–nanosphere:PLA and (e) Au–nanorod:PMMA plates. Scanning-SAXS image of a cross-section through a melt-pressed (f) Au–nanosphere:PLA plate with 5.1 × 10^–3^ wt % Au and (g) Au–nanorod:PMMA plate with 5.0 × 10^–2^ wt % Au. The image combines the average scattering intensity, the oriented intensity, and the preferred orientation angle according to the color wheel legend. It uses a hue-saturation-value representation where a high density of oriented structures corresponds to bright colors, whereas a high density of isotropically scattering structures appears white. Low scattering intensity is represented by black areas. For the nanospheres, no preferred orientation is found, in agreement with their symmetrical geometry. The high scattering intensity and anisotropy appearing as turquoise bands at the sample edges are caused by edge scattering. For the nanorods, a uniform distribution throughout the nanocomposite is revealed and a preferential orientation along the plate-cross section and thus along the filament extrusion direction is identified. (h) Schematic of 3D printing illustrated by (i) Au–nanosphere:PLA Swedish Foundation for Strategic Research logotype coins with different Au loading and (j) an Au–nanorod:PMMA Chalmers University of Technology logotype. The white specimens correspond to objects printed using the matrix polymer only.

We have investigated the impact of surfactant/stabilizer molecules in detail^30^ and found that the cationic surfactants CTAB, CTAC, and TOAB distinctly decelerate the hydrogen sorption rate due to a combination of electronic and steric effects that prevent H_2_ dissociation (Figure 3b). Interestingly, we also found that polymeric surfactants, such as PVP, have the opposite effect and instead accelerate hydrogen sorption. To investigate the underlying reasons, we carried out a combined experimental and first-principles calculations investigation of the impact of 30 nm thin polymer coatings comprised of PMMA and polytetrafluoroethylene (PTFE) films on nanofabricated Pd and PdAu alloy nanoparticles. It revealed a generic effect of accelerated H_2_ sorption in the presence of polymers, as the Pd–polymer interaction reduced relevant activation barriers.^1,72^ Furthermore, our work revealed that the optical contrast generated by hydrogen sorption is increased when nanoparticles are embedded in a polymer due to its high refractive index and that the LoD thereby can be lowered significantly.^1^ As the final beneficial aspect of a polymer coating, we found that molecular filtering can be achieved, through which surface poisoning by CO, NO_2_, CH_4_, and CO_2_ can be eliminated (Figure 3c).^1^ Furthermore, we found that different polymers, depending on their interaction strength with the metal and the FFV, respectively, exhibit different levels of sensor response acceleration and filtering ability and that the application of rationally selected polymer multilayers can maximize sensor performance in these respects with a bilayer of 30 nm PTFE and 35 nm PMMA as the champion system.^1^

**Figure 5:**
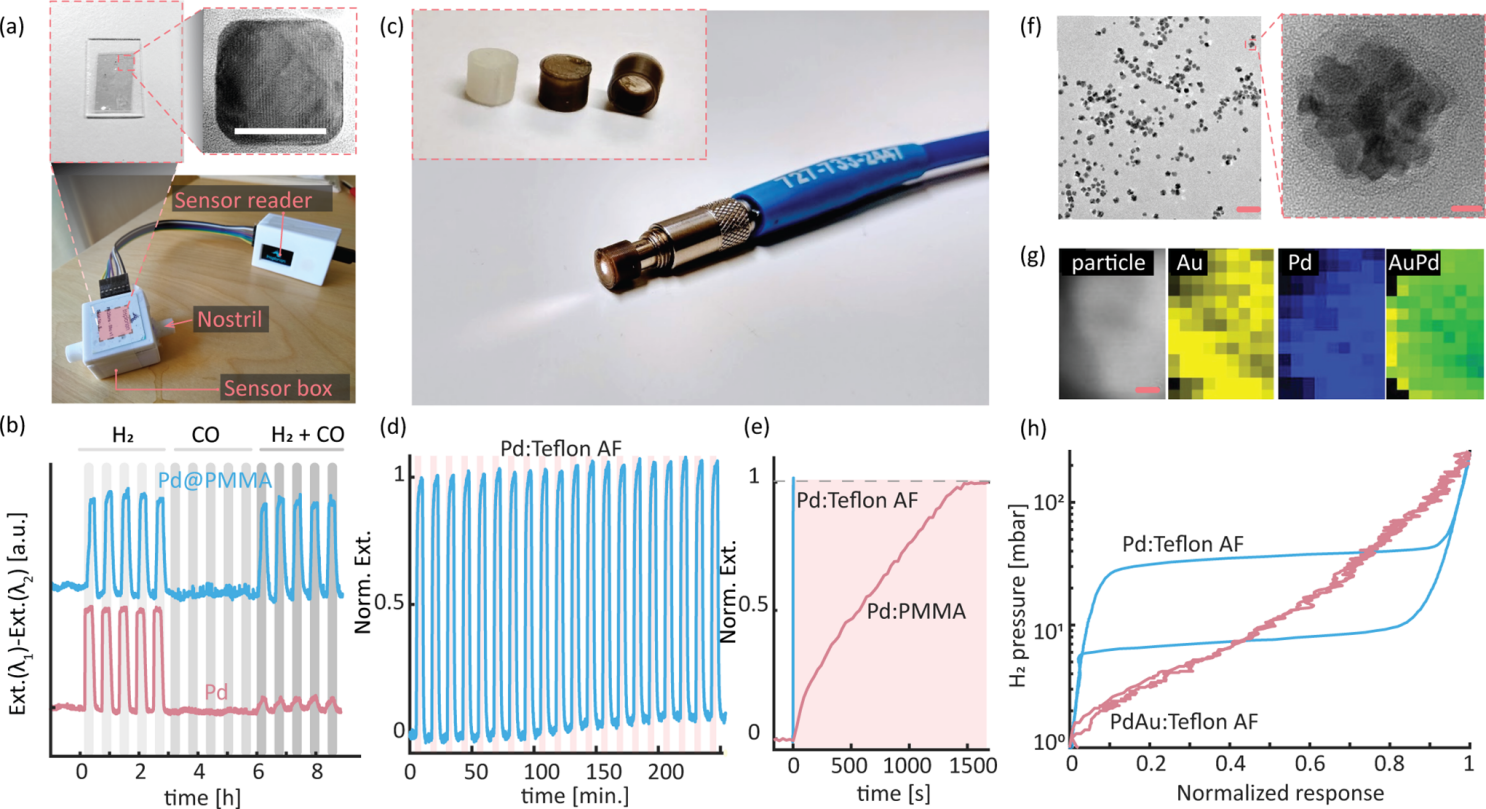
Bulk-processed PMMA and Teflon AF plasmonic plastic H_2_ sensors. (a) Photograph of the sensor device prototype. Inset depicts a 100 μm thick melt-pressed Pd nanocube:PMMA sensor plate with a loading of 0.03 wt % Pd and a TEM image of a Pd nanocube taken after the entire processing sequence (scale bar 20 nm). (b) Sensor response of the Pd nanocube:PMMA plate (blue) and neat Pd nanocubes drop cast onto fused silica support (red) upon exposure to cycles of 10% H_2_, 0.5% CO, and 10% H_2_ + 0.5% CO in synthetic air. Note the resistance to CO deactivation provided by the PMMA matrix. (c) Photograph of a 3D-printed optical fiber sensor comprised of 0.02 wt % Pd:PMMA nanocomposite mounted on an SMA-905 fiber optic connector. Inset depicts neat PMMA (bright) and Pd-loaded PMMA (dark) sensor caps. (a–c) Adapted from ref (3). Copyright 2020 American Chemical Society. (d) 3D-printed Pd:Teflon AF fiber optic sensor cap response to cyclic exposure to 4 vol % H_2_ in synthetic air. Adapted from ref (4). Copyright 2021 American Chemical Society. (e) Response time comparison of a Pd nanocube:PMMA and Pd:Teflon AF sensor for a 4 vol % H_2_ pulse in synthetic air. (f) TEM images of 20 nm colloidal Pd_70_Au_30_ alloy nanoparticles (left panel scale bar 50 nm; right panel scale bar 5 nm). (g) Scanning EDX elemental maps of a colloidal Pd_70_Au_30_ alloy nanoparticle (scale bar 2 nm). (f and g) Adapted from ref (32). Copyright 2021 American Chemical Society. (h) Optical pressure composition isotherms of Pd nanocube:Teflon AF and Pd_60_Au_40_:Teflon AF, revealing the targeted hysteresis-free response of the alloy system (see the SI for details on synthesis and compounding of Pd_60_Au_40_:Teflon AF).

**Figure 6:**
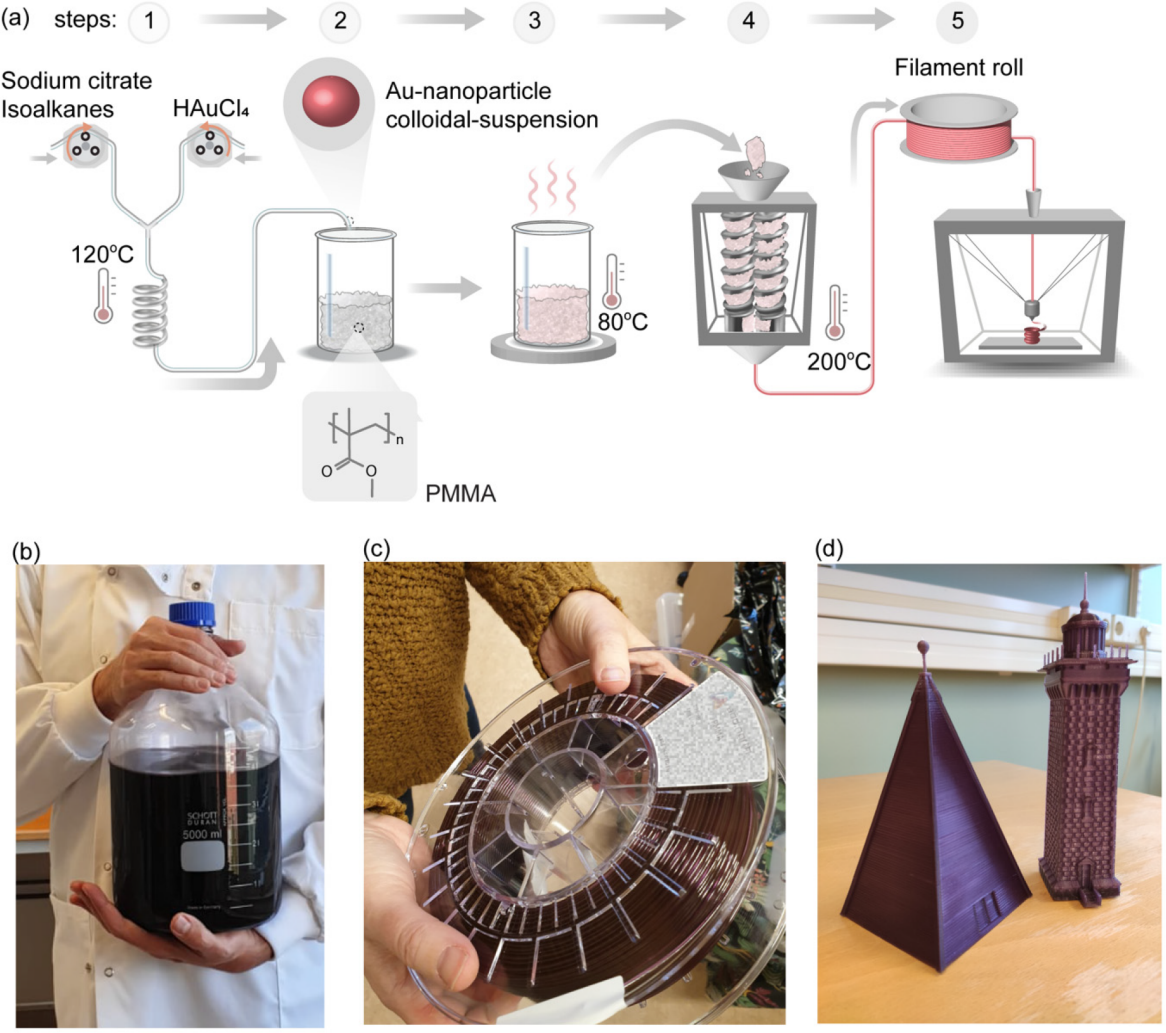
Upscaling of plasmonic plastic production. (a) Processing schematic. (1) Flow synthesis of citrate-stabilized Au nanoparticles. (2) Mixing of the Au nanoparticle dispersion with PMMA powder. (3) Drying of the Au nanoparticle:PMMA slurry. (4) Filament extrusion (note that the initial melt compounding, extrusion, and pelletizing steps have been omitted for clarity). (5) FDM 3D printing. (b) Photograph of the Au nanoparticle suspension. (c) Photograph of the extruded filament. (d) Photographs of 16 and 20 cm tall FDM 3D-printed miniature versions of the Vinga beacon and lighthouse from the Göteborg archipelago in Sweden. See the SI for details on synthesis, compounding, and 3D printing.

## 5 Plasmonic Plastics for Additive Manufacturing of 3D Plasmonic Objects

To conceptually develop fully functional plasmonic plastic materials for additive manufacturing, we report in this section our unpublished results on Au–nanosphere:poly(lactic acid) (PLA) and Au–nanorod:PMMA plasmonic plastic nanocomposites (Figure 4 and Figures S1 and S2). These results complement Kool et al.’s reports of a Au nanoparticle:poly(vinyl alcohol) (PVA) nanocomposite that was used to 3D print a plastic analogue of the famous Lycurgus Cup.^73^ We synthesized two types of Au colloidal nanoparticles, 10 nm spheres and 25 nm × 53 nm nanorods (Figure 4a–c), using CTAB as the surfactant and following the methods reported by Inoue et al.^74^ and Niu et al.,^75^ respectively (for details see SI section 1). We then mixed the nanoparticle dispersion with PLA (Au spheres) and PMMA (Au rods) powder. Subsequently, we compounded the dried mixture in a twin-screw microcompounder and extruded filaments, which served as raw material for fused deposition modeling (FDM) 3D printing (Figure 4a and SI section 1). To characterize both the structural and the optical properties of the obtained materials, we prepared sample sets with systematically increased nanoparticle loading and melt pressed extruded filaments into 500 μm thick plates (Figure 4b and 4c). Optical absorbance measurements of the Au–nanosphere:PLA system revealed a single peak at ca. 550 nm whose intensity increased with Au nanosphere loading (Figure 4d) and two peaks for the Au–nanorod:PMMA system that correspond to a short-wavelength transversal and long-wavelength longitudinal LSPR mode, respectively (Figure 4e). Furthermore, this observation provides indirect confirmation that the shape of the nanorods is preserved during processing, as corroborated by TEM images (Figure S3).

To further characterize the structural properties, scanning small-angle X-ray scattering (s-SAXS) experiments were carried out using cross sections of the melt-pressed plates with the highest sphere/rod loading and maps of the scattering intensity and orientation within a *q* range of 3.3–8.6 × 10^–3^ Å^–1^ were created (Figure 4f and 4g). For the isotropic Au nanospheres, no preferential orientation was observed and the particles appear homogeneously distributed, since the scattering intensity (value) appears uniform in each scan position (Figure 4f and Figures S4 and S7a–c). For the anisotropic Au nanorods, a homogeneous scattering intensity (value) and degree of orientation (saturation) in each scan position was observed, meaning that the rods are uniformly distributed in the matrix (Figure S7d–f) Moreover, the system exhibits a uniformly high degree of alignment of the nanorods in the direction of filament extrusion (Figure 4g and Figure S8), as further corroborated by polarization-dependent optical absorbance measurements and simulations (Figures S5 and S6). This is an important result because it indicates that the polarization-dependent optical properties of plasmonic nanocomposite materials can be unlocked by nanoparticle alignment in the composite. To demonstrate that both types of nanocomposites can be used for FDM (Figure 4h), we 3D printed (see SI section 1) different logotypes (Figure 4i and 4j).

## 6 3D-Printed Plasmonic Plastic Hydrogen Sensors

Based on the insights above, we selected PMMA^3^ and Teflon AF^4^ as polymer matrix materials for separate proof-of-principle studies of 3D-printed plasmonic plastic hydrogen sensors. As hydrogen-sensitive plasmonic nanoparticles, we initially selected CTAB-stabilized colloidal Pd nanocubes due to their robust synthesis protocol before changing the surfactant and taking the step to colloidal PdAu alloy nanoparticles synthesized according to protocols we have optimized for H_2_ sensing.^2,32^

Using PMMA, we created a nanocomposite in which the cubic shape of the Pd nanocrystals was well preserved (Figure 5a) and for which s-SAXS analysis revealed a homogeneous distribution and isotropic arrangement of the particles.^3^ Exposure to H_2_ revealed an overall response characteristic for Pd. Furthermore, we found the optical contrast and response times both to be proportional to the Pd nanoparticle loading and thickness of melt-pressed plates, implying a trade-off between optical contrast and temporal response and a corresponding optimal Pd loading and plate thickness combination. Selecting this optimized system, we integrated a melt-pressed 100 μm thick plate into a gas sensor prototype device (Figure 5a). Remarkably, thanks to the low diffusivity of CO in PMMA and its corresponding excellent molecular filtering ability, the sensor exhibited consistent and reliable response to H_2_ even at severe CO poisoning conditions in synthetic air (Figure 5b) and retained its full functionality after 50 weeks at ambient conditions.^3^ A similar test for 3D-printed sensor caps (Figure 5c) yielded a comparable result and thus corroborated the potential of the overall concept.^3^

As the main drawback, we identified the slow response of the Pd:PMMA system for which we identified PMMA’s low FFV as the reason. To address this particular aspect, in our second study,^4^ we selected Teflon AF as the matrix material due to its significantly larger FFV and the ability of fluorinated polymers to aid sensing processes.^1^ A further advantage of Teflon AF is its high glass transition temperature (e.g., *T*_g_ ≈ 160 °C in the case of Teflon AF 1600), which ensures sensor operation at relatively high temperatures if required by a specific application. To this end, we also note that hydrogen sorption into the metal nanoparticles is an activated process, which means that the sensor response will be accelerated at elevated temperatures. Furthermore, we developed a continuous flow synthesis process that yielded Pd nanocubes stabilized by PVP^2^ rather than CTAB, which is important due to PVP’s positive impact on hydrogen sorption kinetics (Figure 3b)^30^ and from the scalability of nanoparticle synthesis perspective. Using a similar processing protocol for these Pd nanocubes and Teflon AF as for the PMMA system, we prepared both melt-pressed plates and 3D-printed sensor caps (using an FDM printer). The resulting plasmonic plastic H_2_ sensors with response times in the 2 s range (Figure 5d) are orders of magnitude faster than for PMMA analogues (Figure 5e) and feature a LoD down to 30 ppm of H_2_ in synthetic air.^4^

As the main limitation, we identified the hysteresis characteristic for Pd, which is undesirable for continuous H_2_ concentration monitoring. To resolve this issue, we optimized a colloidal synthesis protocol for PdAu alloy colloidal nanoparticles, again using PVP as the stabilizing agent.^32^ Detailed characterization revealed excellent tunability of the composition, a homogeneous distribution of the alloyants (Figure 5f and 5g), and hysteresis-free response to H_2_ for Au concentrations above 30 wt %, in excellent agreement with the 2D model systems (Figure 3a).^32^ Compounding a Teflon AF nanocomposite using corresponding Pd_60_Au_40_ colloidal nanoparticles at 0.3 wt % concentration and melt pressing the nanocomposite into 500 μm thick plates thus yielded a hysteresis-free plasmonic plastic hydrogen sensor as our new and unpublished results reveal (Figure 5h). This is important because it demonstrates that plasmonic plastic materials can be engineered based on design rules derived from well-established 2D analogues nanofabricated onto flat surfaces and that very similar H_2_ sensing characteristics can be obtained for the two fundamentally different material systems.

## 7 Scalability of Plasmonic Plastic Material Synthesis, Compounding, and 3D Printing

To demonstrate that plasmonic plastic material preparation is scalable and that kilogram-scale amounts can be produced, we prepared a nanocomposite composed of Au nanoparticles in a PMMA matrix, which was extruded into a 400 m long filament that we used for FDM 3D printing (Figure 6a). Flow synthesis of citrate-capped spherical Au nanoparticles was carried out under hydrothermal conditions using a modified Turkevich method using isoalkanes as the carrier phase (for details see SI section 1 Experimental Methods and Figure S9). The resulting particles had a mean diameter of 11 nm, consistent with the deep purple color of their aqueous dispersion (Figure 6b). A nanocomposite was prepared by first mixing the Au nanoparticle dispersion with 2 kg of PMMA powder. The powder was then compounded into granules containing 0.01 wt % Au nanoparticles, which were subsequently extruded into 400 m of Au:PMMA filament (Figure 6c). The Au:PMMA filament exhibited excellent printability, enabling stable long-term printing with no sign of curled edges, layer separation, or overheating.

Various types of models were FDM 3D printed, including 16 and 20 cm tall miniature models of the Vinga beacon and lighthouse (total weight 86 g) located in the Göteborg archipelago in Sweden (Figure 6d). The models were designed with 1 mm small features, which could be reproduced with a high degree of accuracy, revealing the excellent quality of the Au:PMMA filament. They also exhibited the same deep purple color as the nanoparticles in solution, reflecting the characteristic LSPR of spherical Au nanoparticles at ∼550 nm (Figure 4) and corroborating well-dispersed particles since aggregates would spectrally red shift the LSPR. The quantity and printability of the prepared nanocomposite filament as well as the level of detail of the FDM 3D-printed models is testimony to the scalability and processability of plasmonic plastic materials.

## 8 Conclusions and Outlook

In this Account, we have summarized our recent cross-disciplinary efforts for the rational design, bulk processing, and practical implementation of plasmonic plastic nanocomposite materials as optical H_2_ sensors produced by additive manufacturing. The flow synthesis of nanoparticles with a well-defined shape provides access to the amounts that are required for bulk processing. Compounding of nanoparticles and matrix polymers can be carried out via an initial solution step followed by melt processing to shape the nanocomposites into objects with various form factors. Melt extrusion of nanocomposites provides access to filaments that can be 3D printed via fused deposition modeling (FDM). Melt-processed nanocomposites comprising Pd nanoparticles readily function as H_2_ sensors with a response time as low as 2 s provided a suitable matrix, such as an amorphous fluoropolymer, is chosen to facilitate rapid ingression of the gas. Device integration of the active sensing materials obtained becomes straightforward in the form of, e.g., melt-pressed plates or fiber-optics-compatible 3D-printed sensor caps that feature a LoD down to the 30 ppm range.

In the future, it can be envisaged that master batches of a nanocomposite are prepared that are then diluted during the subsequent melt-processing step to reach the optimal nanoparticle concentration that is desired for a particular application. The use of multicomponent nanocomposites that comprise nanoparticles of different size, shape, and type can be envisaged. This approach may allow the fabrication of sensors that respond to several types of analytes or combine different time responses, thus creating a memory effect where the sensor not only registers the presence of an analyte but also provides information about overall exposure. In addition to ab initio mixing of multicomponent materials, FDM 3D printing of multilayer structures with different types of nanocomposites may facilitate the on-site manufacture of tailor-made sensor units that combine different types of functionalities. Further, it may be feasible to apply additional blocking layers to fluoropolymer-based nanocomposites for H_2_ sensing with a fast response time to improve their selectivity and protect from poisoning or interfering molecular species like CO, H_2_O, SO_*x*_, or NO_*x*_, resulting in both fast and chemically robust plasmonic plastic H_2_ sensors.

## Supporting Information Available

Experimental methods, nanocomposite processing, transmission electron microscopy of Au nanospheres and Au nanorods after synthesis and after compounding, UV–vis spectra of Au nanospheres and nanorods, small-angle X-ray scattering of Au–nanosphere:PLA and Au–nanorod:PMMA, and transmission electron microscopy of flow-synthesized Au nanospheres (PDF)
