# Supplementary material for: Bulk-Processed Plasmonic Plastic Nanocomposite Materials for Optical Hydrogen Detection
Source: Acc Chem Res. 2023 Jun 23;56(13):1850–61. doi: 10.1021/acs.accounts.3c00182 (PMC10324317; doi:10.1021/acs.accounts.3c00182)
Supplement: Supplementary file 1 — ar3c00182_si_001.pdf [file ar3c00182_si_001.pdf]

Supporting Information

Bulk-Processed Plasmonic Plastic Nanocomposite Materials for Optical Hydrogen Detection

Iwan Darmadi[1], Ida Östergren[2], Sarah Lerch[2], Anja Lund[2], Kasper Moth-Poulsen[2,3,4,5], Christian Müller[2*], Christoph Langhammer[1*]*

[1]Department of Physics, Chalmers University of Technology, 412 96 Göteborg, Sweden

[2]Department of Chemistry and Chemical Engineering, Chalmers University of Technology, 412 96 Göteborg, Sweden

[3]Institute of Materials Science of Barcelona, ICMAB-CSIC, 08193, Bellaterra, Barcelona, Spain

[4]Catalan Institution for Research and Advanced Studies ICREA, Pg. Lluís Companys 23, Barcelona, Spain

[5]Department of Chemical Engineering, Universitat Politècnica de Catalunya, EEBE, Eduard Maristany 10–14, 08019 Barcelona, Spain

*Corresponding authors: kmothpoulsen@icmab.es, christian.muller@chalmers.se, clangham@chalmers.se

1. Experimental methods

1.1 Synthesis of Au nanospheres and Au nanorods

The following chemicals were used: Hexadecyltrimethylammonium bromide (CTAB, ≥98.0%Sigma-Aldrich), Hexadecyltrimethylammonium chloride (CTAC, >95% TCI), L-Ascorbic Acid (99% Sigma-Aldrich), Sodium borohydride ($NaBH_4$, ≥ 98.0%), Silver Nitrate ($AgNO_3$, ≥99.0%, Sigma-Aldrich), Gold (III) chloride trihydrate ($HAuCl_4$, ≥ 99% Sigma-Aldrich),

Au nanospheres stabilized with CTAB were synthesized via a method reported elsewhere53. To obtain Au seeds, 0.5 mL of 1 mM $HAuCl_4$ was mixed with 10 mL of 80 mM CTAC solution at room temperature (21 °C). Ice-cold $NaBH_4$ (30 µL, 100 mM) was injected to the mixture under stirring. The solution changed colour from deep yellow to pale brown. Subsequently, the seed solution was used to prepare ≈10 nm nanospheres. Growth solution A contained: 10 mL of 80 mM CTAB and 1 mL of 2 mM $HAuCl_4$ at 40 °C. 100 µL of 100 mM ascorbic acid was added to growth solution A, resulting in a colour shift from pale yellow to clear. Immediately following the colour shift, 300 µL of as synthesized seed solution was transferred to growth solution A under stirring. Solution A was incubated in a water bath at 40 °C for 20 minutes without stirring. Afterwards, the Au nanospheres were centrifuged (20000 rpm) and dispersed in Milli-Q H_2O (18.2 mΩ/cm).

Au nanorods were obtained via the seed-growth method by Niu et al.54. The seed solution was obtained as follows: 125 µL of 10 mM $HAuCl_4$ solution was added to 5 mL of 100 mM CTAB at 30 °C under gentle stirring. Thereafter 0.3 mL of 10 mM ice-cold $NaBH_4$ was injected to the mixture under rapid stirring and the solution was stirred for 2 more minutes. Subsequently, under stirring, 48 µL of seed solution was added to the mixture of 2 mL 10 mM $HAuCl_4$, 320 µL 10 mM $AgNO_3$, 320 µL 100 mM ascorbic acid in 40 mL of 100 mM CTAB solution at 30 °C. Once everything was mixed thoroughly, the mixture was kept undisturbed in the water bath at 30 °C for 2 hours. Afterwards, 30 mL of the mixture was centrifuged for 10 minutes at 12000 rpm. The supernatant was removed, and the residual was dispersed in 30 mL of Milli-Q water. The centrifugation was repeated (12000 rpm, 10 minutes), the supernatant was removed, and the pellet was dispersed in 30 mL of 100 mM CTAB solution at 40 °C. Subsequently, 1.5 mL of 10 mM $HAuCl_4$ and 0.3 mL of 100 mM ascorbic acid were added to the solution under stirring and the mixture was left to incubate at 40 °C for 1 hour while stirring. Finally, the sample was centrifuged at 12000 rpm anddispersed in Milli-Q H_2O.

1.2 Compounding of Au nanospheres:PLA and Au nanorods:PMMA composites

Poly(methyl methacrylate) (PMMA) powder with a weight-average molecular weight Mw = 75 000 g/mol, a polydispersity index (PDI) = 2.8 and a density of 1.2 g/cm3 was obtained from Polyscience Inc. A 3D printing grade of poly(lactic acid) (PLA) granules obtained from Creative Tools, was ground into a fine powder using a Retsch Rotor Mill.

The Au rods:PMMA composites were prepared by pouring the aqueous dispersions of nanoparticles, Pd or Au, onto the PMMA powder, followed by drying at 80 °C overnight. The dry mixtures were compounded for 5 minutes in an Xplore Micro-compounder MC5 at 200 °C,

extruded through a 1.0 mm die and collected onto a conveyer belt at a slow speed, resulting in filaments with a diameter of 1.75 ± 0.1 mm. The Au spheres:PLA composites were prepared with the same procedure, except that they were dried at 50°C to avoid degradation of the PLA, and extruded through a 1.75 mm die.

1.3 3D-printing via fused filament fabrication Au nanospheres:PLA and Au nanorods:PMMA composites

For the fused filament fabrication of the Au nanospheres:PLA composites we used a Massportal Pharaoh XD printer. The printing was carried out with a nozzle size of 0,4 mm, at a nozzle temperature of 210°C, with a build-plate temperature of 60°C and a printing speed of 2000 mm/min. The Au nanorods:PMMA composites were printed at the same conditions but with a printing speed of 1000 mm/min and with a brim consisting of 8 outlines of PLA, to improve bed adhesion.

1.4 PdAu:Teflon synthesis and compounding

The following chemicals were used without further purification: Gold (III) chloride trihydrate ($HAuCl_4$, ≥99.9%, Aldrich), sodium tetrachloropalladate (Na_2PdCl_4, 98%, Aldrich), L-ascorbic acid (≥99%, Sigma-Aldrich), polyvinylpyrrolidione (PVP, average MW~55 000, Aldrich), and ethylene glycol (AnalaR NORMAPUR, VWR).

PdAu nanoparticles were synthesized following the optimized procedure from ref. 53 in the main text. Briefly, a mixture of $HAuCl_4$ and Na_2PdCl_4 salts were dissolved in MilliQ water such that the total concentration of the metals was 20 mM and the molar ratio was $Pd_{60}Au_{40}$. Separately, 37.5 mL of ethylene glycol and 9.5 mL of MilliQ water were added to a round bottom flask and stirred to combine. 1 mL of $Na_2PdCl_4/HAuCl_4$ solution was added to the flask and stirred until the solution was uniformly pale yellow in colour. 1 mL of L-ascorbic acid (100 mM) was added to the stirred solution, resulting in a change to dark brown/black over the course of approximately 30 seconds. Following the colour change, 1 mL of PVP solution (5mg/mL) was added and the solution was stirred for 30 minutes. Acetone was added to the solution to to bring the total volume up to 150 mL and the nanoparticle solution was centrifuged at 5500 rpm for 60 minutes. The supernatant was removed and the PdAu alloy particles were suspended in isopropanol.

Poly[4,5-difluoro-2,2-bis(trifluoromethyl)-1,3-dioxole-co-tetrafluoroethylene] (Teflon AF) with a dioxole content 65 mol %, a density of 1.78 g cm^{-3}, was obtained from Sigma. Teflon AF was ground to a fine powder using a mortar and pestle and 3.56 g were collected in a glass vial. The PdAu alloys in isopropanol were added to the Teflon AF to obtain a 0.3 wt% dispersion and stirred until well distributed. The mixture was heated at 40 ^{o}C overnight to evaporate the isopropanol. The dry mixture was compounded for 5 minutes in an Xplore Micro-compounder MC5 at 250 °C, extruded and collected on a slow-moving conveyor belt. 500 µm thick samples for hydrogen sensing were prepared using a hot press at 240 °C and increasing pressure up to 10 tons over 8 minutes.

1.5 PdAu:Teflon H_2 pressure-composition measurement

The measurement were performed using a vacuum chamber (effective volume ≈ 40 mL, the schematic is available in detail in ref.[1]). The chamber is wrapped with a heating coil and the interior temperature is monitored by a thermocouple in contact with the hydrogen sensor chip. The heating coil and the thermocouple are connected to a Eurotherm thermocontroller (Eurotherm 3216), which regulates the chamber temperature in a feedback-loop manner. The chamber is equipped with two fused-silica viewports (1.33" CF Flange, Accu-Glass), which enable transmission mode optical measurements using a fiber-coupled polychromatic halogen light source (Avantes AvaLight-Hal) and a fixed-grating spectrophotometer (Avantes SensLine AvaSpec-2048XL).

The chamber is connected to a pure 100% hydrogen source at one end and to a turbo vacuum pump at the other end. Hydrogen pressure in the chamber is controlled by either a leak valve. The chamber pressure is monitored by two capacitance pressure gauges (MKS Barathron 626C) of different pressure range. The first manometer is used to monitor 1-1000 mbar pressure, while the second one is for 1-1000 μbar. The pressure-composition isotherm were measured at 30°C. For the descriptor, we use ΔExt where the procedure is described in detail in ref.[2]

1.6 Upscale synthesis of Au nanoparticles

The Au NPs were synthesized under hydrothermal conditions, by continuous-flow liquid-liquid using an isoalkanes as carrier phase. The flow synthesis of citrate-capped Au NPs was adapted from the modified Turkevich method. The synthesis was performed using a flow system featuring two peristaltic pumps, a coiled reactor (10 mL), which was kept at constant temperature (120°C). A back-pressure regulator was introduced after the coiled reactor. Reagents were pumped in perfluoroalkoxy (PFA) tubes (i.d. 1 mm). An aqueous solution of sodium citrate 10 mM and citric acid 2 mM (Solution 1), gold chloride trihydrate solution 1 mM (Solution 2) and isoalkane (Solution 3) were mixed in the ratio of 1:1:5, respectively. Citrate and Au-precursor solution were interfaced by a T-junction, whose outlet was connected to a glass capillary, entering a second T-junction. Droplets produced by the capillary created a segmented flow when the carrier phase was infused into the second T-junction using a peristaltic pump. The residence time in the coiled reactor was calculated to 2 min. The resulting purple aqueous mixture containing the Au nanoparticles were collected from the biphasic system in a separatory funnel and directly used in the production of composite.

1.7 Upscaled compounding of Au:PMMA composite

A nanocomposite was prepared by wet mixing the Au nanoparticle dispersion with PMMA powder (see ref3 for details about the polymer), followed by slow drying of the resulting slurry under vacuum at 80 °C.

The dried powder was compounded in a Coperion ZSK 26 twin screw extruder at 200 °C, extruded into a water bath and then directly fed into a Scheer pelletizer to produce nanocomposite granules containing 0.01 wt% Au nanoparticles.

400 m of Au:PMMA filament with a diameter of 1.75 mm (total weight 1.5 kg) were extruded at 200 °C in a filament production line at Add:north, Ölsremma.

FDM 3D printing was carried out using a Massportal Pharaoh XD20 at a printing speed of 3600 mm min^{-1} which was reduced to 500 mm min^{-1} for the top scepter of the beacon and lighthouse, a nozzle diameter of 0.4 mm, and with a nozzle temperature of 260 °C. The bed temperature was 110 °C for the first printing layer to achieved good bed adhesion, which was then decreased to 60 °C for solidification and to avoid warping.

1.8 Transmission electron microscopy

All nanoparticles and nanocomposites were imaged by using a Tecnai T20 TEM microscope, operating at 200 kV with a LaB6 gun and a Titan 80-300 TEM microscope, operating at 300 kV with a field emission gun. Pd nanocubes, Au nanospheres and Au nanorods samples were prepared on pure carbon 200 mesh copper grid. Pd nanocubes, Au nanospheres and Au nanorods nanocomposite samples were prepared on Lacey 400 mesh copper grid.

1.9 UV-vis spectroscopy

The UV-visible absorbance spectra were obtained with a Cary 5000 spectrophotometer within the range 350–800 nm. For polarization-dependent measurements, a Glan-Taylor polarizer accessory (PGT-S1V) from Harrick Scientific Products, Inc. was used.

1.10 Scanning Small Angle X-Ray Scattering (scanning-SAXS)

Scanning SAXS measurements were carried out at the cSAXS beamline of the Swiss Light Source (SLS) at the Paul Scherrer Institute (PSI) in Villigen, Switzerland. The samples were scanned at an energy of 11.2 keV and the beam size was 40 × 40 µm and 25 × 10 µm for the Pd- and Au-containing composites, respectively. The sample-to-detector distance was 2.16 m. A Pilatus 2M detector was used to acquire small-angle scattering patterns. The step sizes used were 40 × 40 µm and 25 × 25 µm, respectively, while the applied exposure time was 0.2 s. Data processing was carried out using the “cSAXS scanning SAXS package” developed by the CXS group, Paul Scherrer Institute, Switzerland.[3] The size determination was performed using the SASView 4.2.0 software.

2. Nanocomposite processing

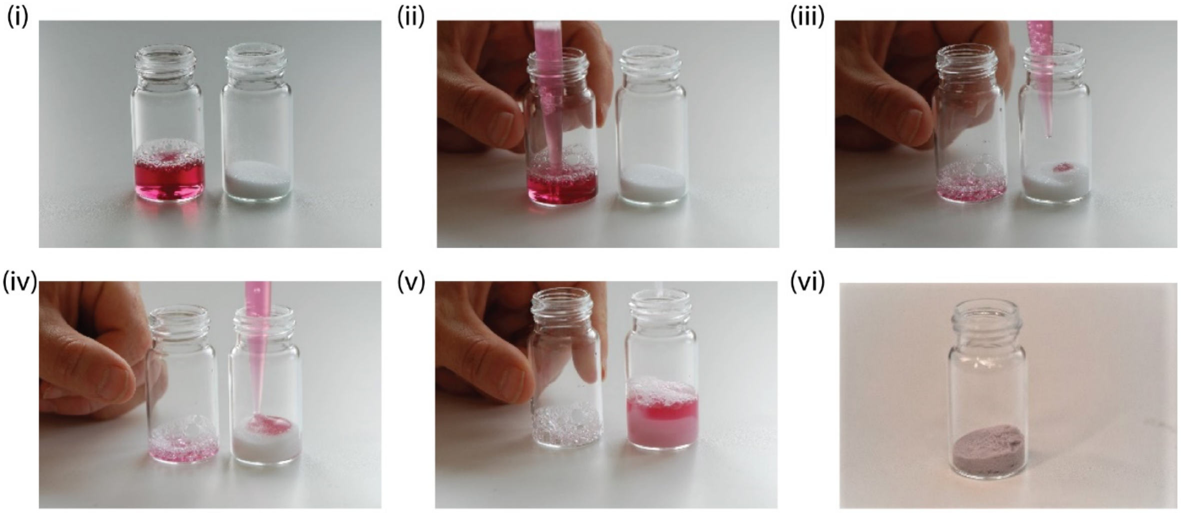

Figure S1. *General scheme for nanoparticle-polymer composite process sequence: (i) Nanoparticle-dispersion and polymer powder preparation, (ii)-to-(v) Nanoparticle-dispersion transfer to polymer powder holder using pipette and (vi) Solvent evaporation by heating leaving a dried mixture of nanoparticles and polymer powder .*

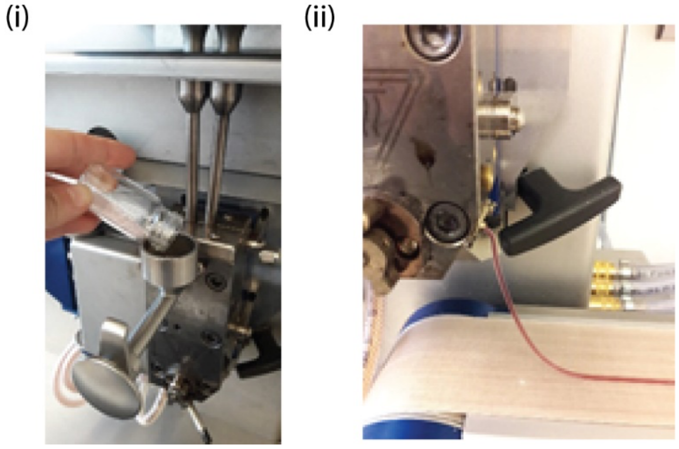

Figure S2. *Nanoparticle-polymer composite extrusion process: (i) Nanoparticles and polymer powder dry mixture fed into twin-screw extruder for compunding. (ii) Extruded nanoparticle-polymer composite filament collected by slow-speed conveyor belt.*

3. Transmission electron microscopy of Au nanospheres and Au nanorods after synthesis and after compounding

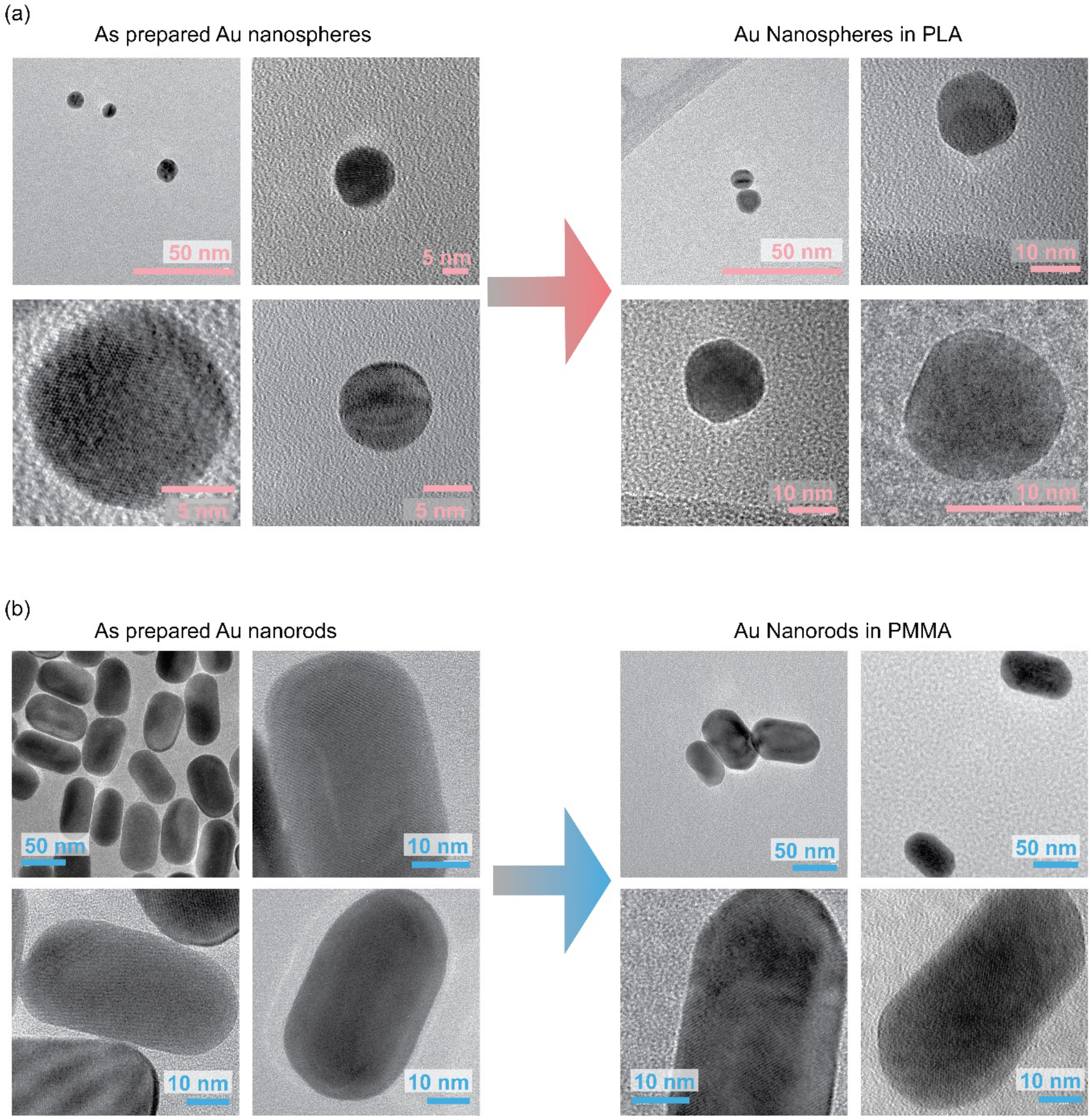

Figure S3. *High resolution TEM of representative Au nanospheres (a) and Au nanorods (b) after colloidal synthesis and after compounding process.*

4. UV-vis spectra of Au nanospheres and nanorods

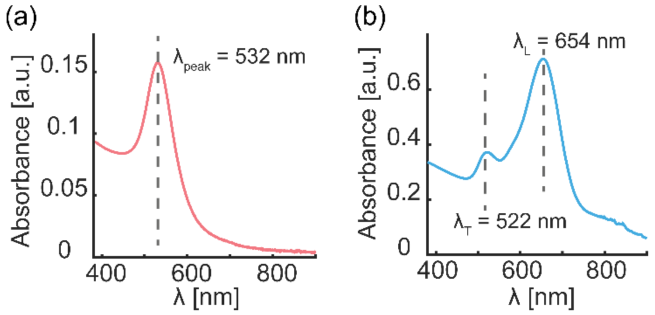

Figure S4. *UV-Visible absorbance spectra of the colloidal suspensions of as-synthesized (a) Au-nanospheres and (b) Au-nanorods. The Au-nanosphere suspension shows a plasmonic peak at λ = 532 nm, while the Au-nanorod suspension exhibits two peaks, which correspond to transversal (at λ_T = 522 nm) and longitudinal plasmon mode (at λ_L = 654 nm).*

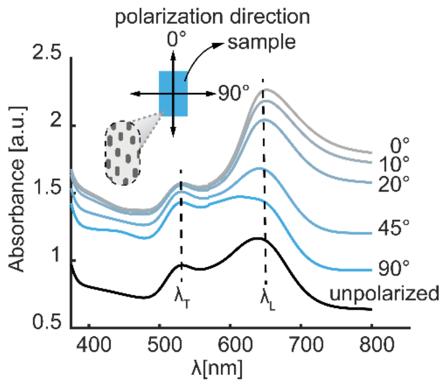

Figure S5. *Optical absorbance spectra of Au-nanorod:PMMA plate with 5.0 × 10^{-2} wt.% nanorod loading and 500 µm thickness, measured at different polarization angles. When polarization is parallel to the sample plate and thus nanorod preferred orientation according to the c-SAXS analysis (0°), the longitudinal mode peak (λ_L) is more pronounced than the transversal mode peak (λ_T), corroborating that most of the Au nanorods are aligned in parallel.*

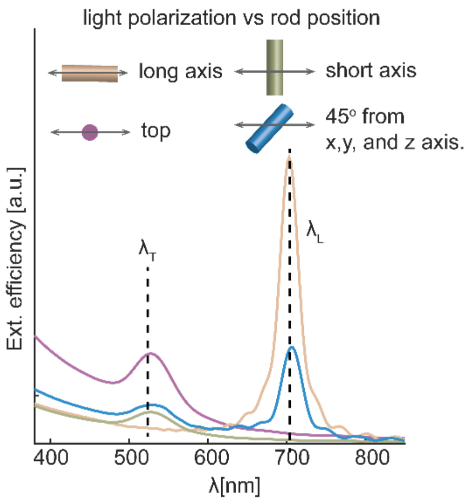

Figure S6. *Simulated extinction efficiencies of an Au nanorod (diameter = 25 nm, length = 56 nm) for differently polarized incident plane waves. Light polarization along the nanorod long axis excites a longitudinal plasmon mode (λ_L), while light polarized along the nanorod short axes induces a transversal plasmon mode (λ_T). 45°-polarization excites both longitudinal and transversal plasmon modes.*

We used FDTD Solutions (Lumerical) to calculate the polarization-dependent optical extinction spectra of an Au nanorod. It is modelled with a long axis of 56 nm and short axes of 25 nm. The dielectric function of Au is taken from the literature.[4] The rod is embedded in a dielectric medium with the dielectric function of PMMA obtained from ref.[5] The structures are illuminated by a plane wave and extinction is collected and analysed in terms of resonance position.

5. Small angle X-ray scattering of Au-nanosphere:PLA and Au-nanorod:PMMA

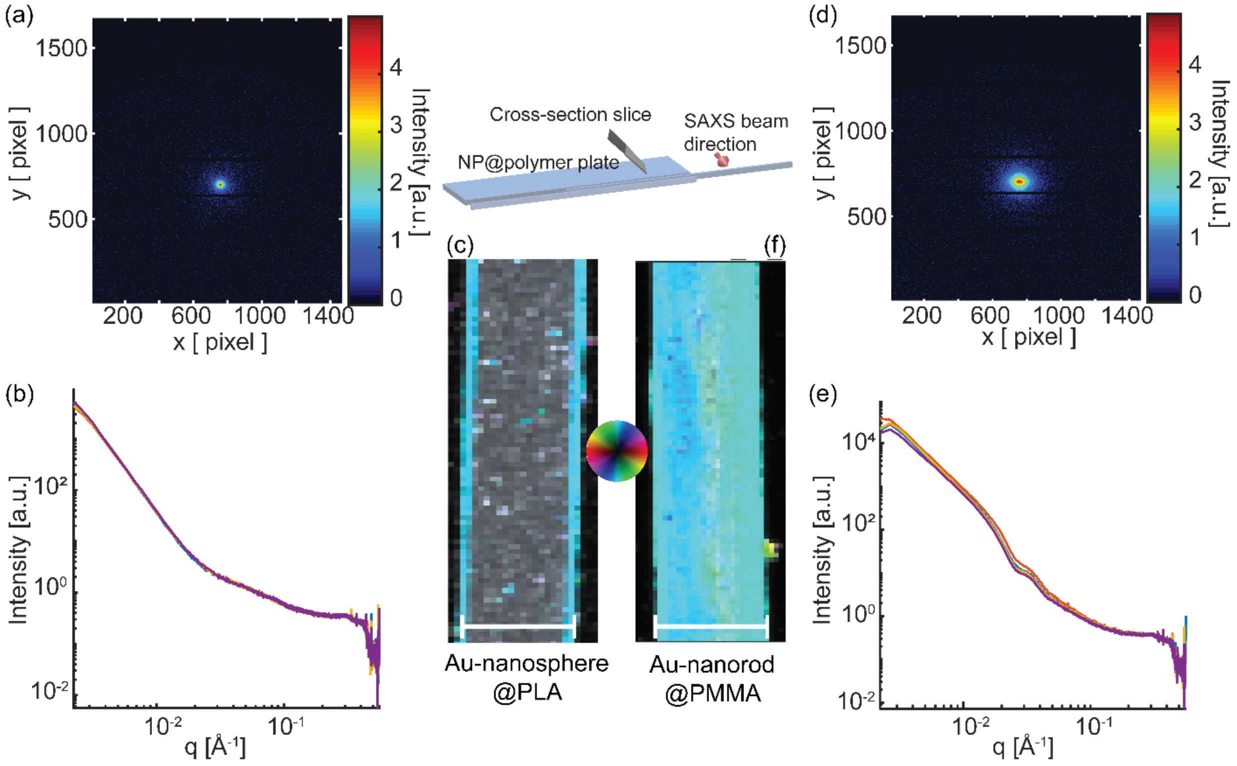

Figure S7. *(a,d) 2D detector scans and (b,e) scattering curves obtained from the corresponding (c) Au-nanosphere:PLA and (f) Au-nanorod:PMMA cross-section slice, as indicated in the schematic depiction. Differently coloured lines correspond to scattering curves obtained at different positions in the sample. The cross-section slice is 200 μm thick and the SAXS-beam direction with respect to the slice cut orientation is illustrated in the schematic. Scale bar is 500 μm. The image combines the average scattering intensity, the oriented intensity and the preferred orientation angle (if there is any), according to the colour wheel, using a hue-saturation-value representation. A high density of oriented structures shows up as bright colours, whereas a high density of isotropically scattering structures will show up in white. Low scattering intensity would be represented by black areas. The differently coloured lines in (b) and (e) correspond to scattering signals from representative points in (c) and (f), respectively.*

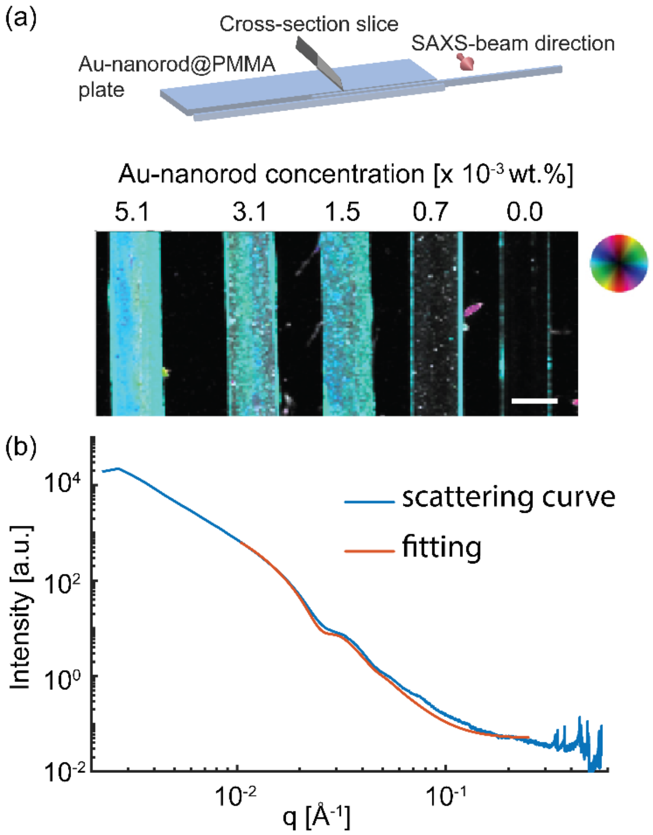

Figure S8. *(a) Scanning SAXS measurements of Au-nanorod:PMMA (from left to right: (i) 5.1, (ii) 3.1, (iii) 1.5, (iv) 0.7, and (v) 0 × 10^{-3} wt.% Au concentration, respectively). The cross-section thickness is 200 µm. The cross-section cut of the plate and the relative SAXS-beam direction is illustrated. The image combines the average scattering intensity, the oriented intensity and the preferred orientation angle (if there is any), according to the colour wheel, using a hue-saturation-value representation. A high density of oriented structures shows up as bright colours, whereas a high density of isotropically scattering structures will show up in white. Low scattering intensity would be represented by black areas. The nanoparticles are aligned in the same direction regardless of their concentration thus the matching colour of the samples. The increasing colour intensity is related to the increasing amount of signal from aligned nanoparticles. (b) Averaged small-angle X-ray scattering curve of 80 representative points of the highest-concentration Au-nanorod:PMMA (blue curve) with the corresponding cylinder fit (red curve). The signal has been corrected for neat PMMA background.*

6. Transmission electron microscopy of flow-synthesized Au nanospheres

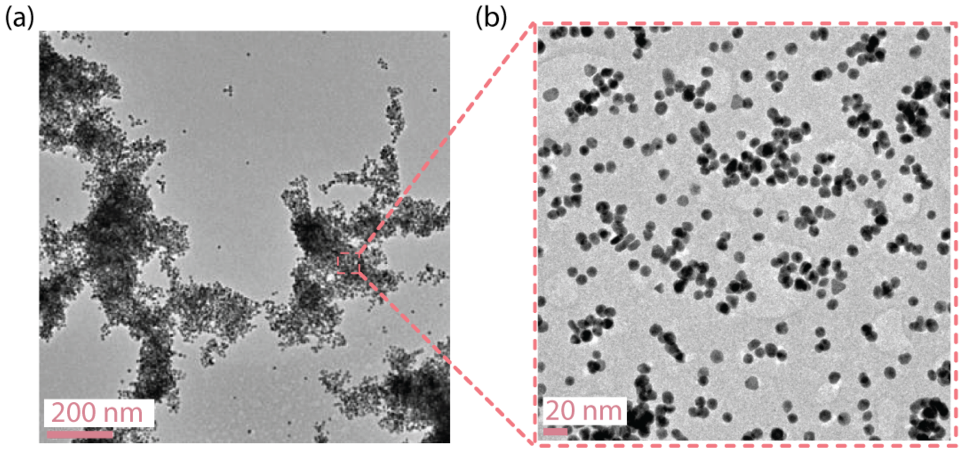

Figure S9. *TEM images of citrate-capped Au nanoparticles produced by continuous-flow liquid-liquid synthesis method. The mean particles diameter is 10.6 ± 9% nm.*

7. References

(1) Darmadi, I. Polymer-Nanoparticle Hybrid Materials for Plasmonic Hydrogen Detection, Chalmers University of Technology, 2021.

(2) Darmadi, I.; Stolaś, A.; Östergren, I.; Berke, B.; Nugroho, F. A. A.; Minelli, M.; Lerch, S.; Tanyeli, I.; Lund, A.; Andersson, O.; et al. Bulk-Processed Pd Nanocube–Poly(Methyl Methacrylate) Nanocomposites as Plasmonic Plastics for Hydrogen Sensing. *ACS Appl. Nano Mater.* **2020**, *3* (8), 8438–8445.

(3) Bunk, O.; Bech, M.; Jensen, T. H.; Feidenhans'L, R.; Binderup, T.; Menzel, A.; Pfeiffer, F. Multimodal X-Ray Scatter Imaging. *New J. Phys.* **2009**, *11* (12), 123016.

(4) Weaver, J. H.; Frederikse, H. P. R. Optical Properties of Selected Elements. In *CRC Handbook of Chemistry and Physics*; 2019; p Section 12 (Internet edition).

(5) Fujiseki, T.; Fujimoto, S.; Campoy-Quiles, M.; Alonso, M. I.; Murakami, T. N.; Miyadera, T.; Fujiwara, H. Organic Semiconductors. In *Spectroscopic Ellipsometry for Photovoltaics vol. 2*; 2018; p 455.
